# Supplementary material for: A rapid and reliable liquid chromatography/mass spectrometry method for SARS-CoV-2 analysis from gargle solutions and saliva
Source: Anal Bioanal Chem. 2021 Aug 24;413(26):6503–11. doi: 10.1007/s00216-021-03614-y (PMC8383918; doi:10.1007/s00216-021-03614-y)
Supplement: ESM 1 — (DOCX 17 kb) [file 216_2021_3614_MOESM1_ESM.docx]

**Supporting Information**

**A Rapid and Reliable Liquid Chromatography/Mass Spectrometry Method**

**for SARS-CoV-2 Analysis from Gargle Solutions and Saliva**

Marc Kipping ^1,2*^, Dirk Tänzler ^1,2^ and Andrea Sinz ^1,2^*

^1^ Department of Pharmaceutical Chemistry & Bioanalytics, Institute of Pharmacy, Kurt-Mothes-Str. 3, D-06120 Halle (Saale), Germany; ^2^ Center for Structural Mass Spectrometry, Kurt-Mothes-Str. 3, D-06120 Halle (Saale), Germany

*Address correspondence to:

Dr. Marc Kipping, Tel: +49-345-5525228, Fax: +49-345-5527026,

Email: [marc.kipping@pharmazie.uni-halle.de](mailto:marc.kipping@pharmazie.uni-halle.de)

Prof. Dr. Andrea Sinz, Tel: +49-345-5525170, Fax: +49-345-5527026,

Email: [andrea.sinz@pharmazie.uni-halle.de](mailto:andrea.sinz@pharmazie.uni-halle.de)

**Content**

The Supporting Information comprises one Excel file (SARS_CoV_2_LC_MRM.xlsx) with

- response areas and peak heights extracted from LC/MRM raw files (TargetLynx, Waters)
- calculations
- complete list of MRM transitions
